# Cardiovascular Disease Risk Factors in US Adults With Vision Impairment

**DOI:** 10.5888/pcd19.220027

**Published:** 2022-07-21

**Authors:** Isabel Mendez, Minchul Kim, Elizabeth A. Lundeen, Fleetwood Loustalot, Jing Fang, Jinan Saaddine

**Affiliations:** 1 Division of Diabetes Translation, National Center for Chronic Disease Prevention and Health Promotion, Centers for Disease Control and Prevention, Atlanta, Georgia; 2 University of Illinois College of Medicine, Peoria, Illinois; 3 Division for Heart Disease and Stroke Prevention, National Center for Chronic Disease Prevention and Health Promotion, Centers for Disease Control and Prevention, Atlanta, Georgia

## Abstract

**Introduction:**

Adults with vision impairment (VI) have a higher prevalence of cardiovascular disease (CVD) compared with those without VI. We estimated the prevalence of CVD and CVD risk factors by VI status in US adults.

**Methods:**

We used nationally representative data from the 2018 National Health Interview Survey (N = 22,890 adults aged ≥18 years). We estimated the prevalence of self-reported diagnosis of CVD (coronary heart disease [including angina and myocardial infarction], stroke, or other heart disease) by VI status. We used separate logistic regression models to generate adjusted prevalence ratios (aPRs), controlling for sociodemographic covariates, for those with VI (reference group, no VI) for CVD and CVD risk factors: current smoking, physical inactivity, excessive alcohol intake, obesity, hypertension, high cholesterol, and diabetes.

**Results:**

Overall, 12.9% (95% CI, 12.3–13.5) of the sample had VI. The prevalence of CVD was 26.6% (95% CI, 24.7–28.6) in people with VI versus 12.2% (95% CI, 11.7–12.8) in those without VI (aPR = 1.65 [95% CI, 1.51–1.80]). Compared with adults without VI, those with VI had a higher prevalence of all risk factors examined: current smoking (aPR = 1.40 [95% CI, 1.27–1.53]), physical inactivity (aPR = 1.14 [95% CI, 1.06–1.22]), excessive alcohol intake (aPR = 1.29 [95% CI, 1.08–1.53]), obesity (aPR = 1.28 [95% CI, 1.21–1.36]), hypertension (aPR = 1.29 [95% CI, 1.22–1.36]), high cholesterol (aPR = 1.21 [95% CI, 1.14–1.29]), and diabetes (aPR = 1.54 [95% CI, 1.38–1.72]).

**Conclusion:**

Adults with VI had a higher prevalence of CVD and CVD risk factors compared with those without VI. Effective clinical and lifestyle interventions, adapted to accommodate VI-related challenges, may help reduce CVD risk in adults with VI.

**Summary**

**What is already known on this topic?**

A strong relationship exists between cardiovascular health and eye health, and research indicates that adults with vision impairment (VI) have a higher prevalence of cardiovascular disease (CVD) compared with those without VI.

**What is added by this report?**

We documented differences in prevalence of CVD risk factors between people with and without VI.

**What are the implications for public health practice?**

A better understanding of the relationship between VI status and CVD risk factors may aid in the prevention and management of CVD in people with VI.

## Introduction

Cardiovascular disease (CVD), including heart disease, stroke and vascular disease, is a major cause of illness and death in the US, claiming 800,000 lives each year (1). CVD contributes $363 billion annually in health care costs and lost productivity (1).

CVD can be prevented or delayed through lifestyle modifications to control or manage risk factors. Approximately 34% of deaths from heart disease could be prevented by modifying key risk factors (2). The American Heart Association (AHA) promotes Life’s Simple 7 (LS7) (3), which identifies and quantifies 7 factors that influence cardiovascular health (smoking status, physical activity, body weight, diet, blood pressure, cholesterol, and blood glucose), with higher LS7 scores associated with better cardiovascular health and lower risk of all-cause and CVD mortality (1,4).

A strong connection between cardiovascular health and eye health has been noted (5); they share risk factors such as older age, current smoking, high blood glucose, and hypertension. One study found that adults aged 40 years or older who had better cardiovascular health had lower odds of ocular diseases such as age-related macular degeneration (AMD), diabetic retinopathy, cataract, and glaucoma (5). Research has also shown that compared with adults without vision impairment (VI), those with VI have a higher prevalence of CVD, contributing to increased mortality risk among the 7 million Americans with VI (6,7). A study of US adults aged 65 years or older found that compared with people without VI, people with VI had a higher prevalence of 13 self-reported chronic conditions, including heart disease and stroke (7). 

Although studies have examined the relationship between VI and CVD (7–9), less is known about differences in prevalence of CVD risk factors between people with and without VI. Better understanding the relationship between VI and CVD risk factors may aid in prevention and management of CVD among those with VI. Our objective was to assess the relationship between VI and CVD risk factors in US adults.

## Methods

We analyzed publicly available, de-identified data from the sample adult core questionnaire of the 2018 National Health Interview Survey (NHIS). The NHIS is an annual, cross-sectional, in-person household interview survey of US noninstitutionalized civilians in all 50 states and the District of Columbia. The NHIS is among the primary data collection programs of the Centers for Disease Control and Prevention’s (CDC’s) National Center for Health Statistics and is a principal source of information on health outcomes, risk factors, and behaviors in the US. The NHIS uses a complex probability sampling strategy to select households and individuals, and estimates are weighted to represent the US adult civilian population. Respondents provided oral consent before participation, and the survey was approved by CDC’s Research Ethics Review Board and the US Office of Management and Budget.

### Study sample

The sample adult component contained data for 25,417 respondents aged 18 years or older and had an unconditional final response rate of 53.1% in 2018 (10). We excluded pregnant people and those missing data on self-reported CVD, CVD risk factors, and VI (n = 2,527), yielding a final analytic sample of 22,890 adults.

### Measures

Our exposure was self-reported VI and was characterized as an affirmative response to the question: “Do you have difficulty seeing, even when wearing glasses?” The outcomes we investigated were self-reported CVD and 7 CVD risk factors. Self-reported CVD was ascertained by asking whether the respondent had ever been told by a doctor or other health professional that they had any of the following conditions: coronary heart disease, angina/angina pectoris, heart attack/myocardial infarction, stroke, or any kind of heart condition or heart disease. Using AHA’s LS7 as a framework, we selected 7 self-reported CVD risk factors from the NHIS to examine cardiovascular health: current smoking, physical inactivity, excessive alcohol intake, obesity, hypertension, high cholesterol, and diabetes. Because NHIS does not regularly collect dietary data as part of its core survey content, dietary data were not collected in 2018 and could not be used; because the consumption of alcohol has complex effects on cardiovascular health, we included excessive alcohol intake in place of poor diet as a CVD risk factor (11). The self-reported CVD risk factors were separated into 2 categories: 1) risk behaviors: current smoking, physical inactivity, and excessive alcohol intake; and 2) health conditions: obesity, hypertension, high cholesterol, diabetes. The 3 risk behaviors were characterized as: current smoker (defined as those who had smoked more than 100 cigarettes in their lifetime and now smoke every day or some days), physical inactivity (defined as performing <10 minutes per week of light, moderate, or vigorous leisure-time physical activities), excessive alcohol intake (defined as consuming ≥12 drinks in their lifetime and >14 drinks/week in past year [for men] or >7 drinks/week in past year [for women]). Alcohol intake for the full adult sample was used for analyses; however, in the US the Minimum Legal Drinking Age (MLDA) has been 21 years since 1984 (12). The 4 health conditions were obesity (body mass index >30 kg/m^2^, calculated using self-reported height and weight) and self-reported hypertension, high cholesterol, and diabetes, which were defined as an affirmative response to the question of whether the respondent had ever been told by a doctor or other health professional that they had hypertension or high blood pressure, high cholesterol, or diabetes or sugar diabetes, respectively. The NHIS does not directly measure blood pressure or collect biospecimens, so self-reported factors were used as proxy assessments.

Sociodemographic characteristics were age, sex, race and ethnicity (non-Hispanic Black, Hispanic, non-Hispanic White, and other racial/ethnic groups), education (less than high school, high school/general educational development, more than high school), marital status (married/domestic partnership, not married [including widowed, divorced, separated, or never married]), employment status (work for pay at job/business, not working for pay), health insurance (public, private, both, none), and family income-to-poverty threshold ratio (<1, 1 to <2, ≥2) based on the US Census Bureau federal poverty thresholds (https://www.census.gov/data/tables/time-series/demo/income-poverty/historical-poverty-thresholds.html).

### Statistical analysis

Descriptive characteristics of the study population were tabulated, stratified by VI status. We used separate logistic regression models to generate adjusted prevalence ratios (aPRs) for those with VI (reference: no VI) for CVD and the 7 CVD risk factors. Models for each outcome controlled for age (as a continuous variable), sex, race and ethnicity, education level, marital status, employment status, income-to-poverty ratio, and health insurance. We examined the effect modification of the relationship between VI, CVD, and the 7 CVD risk factors by calculating the aPR for each age group (18–44 y, 45–64 y, ≥65 y) derived from a model that included an interaction term between VI and age group. We used χ^2^ tests to examine whether the prevalence of VI varied by sociodemographic characteristics (differences considered significant at *P* < .05). We also determined the distribution of respondents by the number of CVD risk factors and VI status. All analyses accounted for complex survey design and sampling weights. Weighted analyses were performed using STATA version 16 (StataCorp LLC).

## Results

Nearly half of adults in this study were aged 18 to 44 years (46.3%), and most were non-Hispanic White (63.9%), had more than a high school education (64.7%), were married (60.3%), worked for pay at a job or business (63.0%), had an income-to-poverty ratio of 2 or more (73.3%), and had private health insurance (54.6%) (Table 1). Overall, 12.9% (95% CI, 12.3–13.5) of adults had self-reported VI. Compared with adults without VI, those with VI tended to be older (≥45 years), female, non-Hispanic Black, not married, and not working for pay and to have a high school education or less, an income-to-poverty ratio of <1 or 1 to <2, and public health insurance. Overall, the prevalence of CVD among adults was 14.1% (95% CI, 13.5–14.7) (Table 2). Prevalence of CVD was 26.6% (95% CI, 24.7–28.6) in respondents with VI and 12.2% (95% CI, 11.7–12.8) in those without VI (prevalence ratio [PR] = 2.18 [95% CI, 2.00–2.37]). In unadjusted analyses, respondents with VI had a significantly higher prevalence of CVD and 6 of the 7 CVD risk factors. After adjusting for sociodemographic factors, compared with adults without VI, those with VI had a higher prevalence of CVD (aPR = 1.65 [95% CI, 1.51–1.80]) and all 3 CVD risk behaviors: current smoking (aPR = 1.40 [95% CI, 1.27–1.53]), physical inactivity (aPR = 1.14 [95% CI, 1.06–1.22]), and excessive alcohol intake (aPR = 1.29 [95% CI, 1.08–1.53]). Additionally, in adjusted analyses, respondents with VI had a higher prevalence of all 4 self-reported health conditions: obesity (aPR = 1.28 [95% CI, 1.21–1.36]), hypertension (aPR = 1.29 [95% CI, 1.22–1.36]), high cholesterol (aPR = 1.21 [95% CI, 1.14–1.29]), and diabetes (aPR = 1.54 [95% CI, 1.38–1.72]). In models examining effect modification by age group, the aPR was higher for CVD and several CVD risk factors among the younger age groups (18–44 years and 45–64 years) compared with the older age group (≥65 years); however, this effect modification was only significant (*P* < .05) for 3 models (CVD, hypertension, and diabetes) (Table 3). Overall, compared with adults without VI, those with VI had a higher number of CVD risk factors (Table 4). Among those with VI, more than 61% reported having 2 or more CVD risk factors, whereas 40% of those without VI did.

**Table 1:** Selected Characteristics of Adults Aged ≥18 Years, by Self-Reported Vision Status, Study of Cardiovascular Disease Risk Factors in US Adults With Vision Impairment, 2018

Characteristic	All, % (95% CI) (N = 22,890)	Distribution by vision status, % (95% CI)^a^	
		Vision impairment (n = 3,214)	No vision impairment (n = 19,676)
Age, y^b^			
18–44	46.3 (45.3–47.3)	31.1 (28.9–33.3)	48.5 (47.5–49.6)
45–64	33.4 (32.6–34.2)	40.8 (38.7–43.0)	32.3 (31.4–33.1)
≥65	20.3 (19.6–21.0)	28.1 (26.3–29.9)	19.2 (18.4–19.9)
Sex^b^			
Male	48.8 (47.6–49.2)	43.0 (40.8–45.2)	49.7 (48.9–50.5)
Female	51.2 (50.4–51.9)	57.0 (54.8–59.2)	50.3 (49.5–51.1)
Race and ethnicity^b^			
Black, non-Hispanic	11.2 (10.3–12.1)	14.0 (12.2–16.0)	10.8 (9.9–11.7)
Hispanic	16.0 (14.7–17.4)	16.0 (13.9–18.3)	16.0 (14.7–17.4)
Other	8.9 (8.1–9.7)	7.4 (6.0–9.1)	9.1 (8.3–10.0)
White, non-Hispanic	63.9 (62.3–65.5)	62.6 (59.9–65.3)	64.1 (62.5–65.7)
Education level^b^			
Less than high school	11.0 (10.3–11.8)	14.8 (13.2–16.5)	10.4 (9.7–11.2)
High school/GED	24.3 (23.4–25.1)	27.6 (25.6–29.8)	23.8 (22.9–24.7)
More than high school	64.7 (63.6–65.8)	57.6 (55.3–59.9)	65.8 (64.6–66.9)
Marital status^b^			
Married/domestic partnership	60.3 (59.4–61.2)	54.7 (52.5–56.8)	61.2 (60.2–62.1)
Not married^c^	39.7 (38.8–40.6)	45.3 (43.2–47.5)	38.8 (37.9–39.8)
Employment status^b^			
Work for pay at job/business	63.0 (62.1–64.0)	46.7 (44.4–48.9)	65.5 (64.5–66.4)
Not working for pay	37.0 (36.0–37.9)	53.3 (51.1–55.6)	34.5 (33.6–35.5)
Income-to-poverty ratio^b^^,^ d			
<1	10.0 (9.4–10.6)	15.0 (13.5–16.6)	9.2 (8.6–9.8)
1 to <2	16.7 (16.0–17.5)	22.9 (21.1–24.9)	15.8 (15.0–16.6)
≥2	73.3 (72.3–74.4)	62.1 (59.7–64.3)	75.0 (73.9–76.0)
Health insurance^b^			
Public	23.9 (23.0–24.8)	36.2 (33.9–38.5)	22.0 (21.2–22.9)
Private	54.6 (53.5–55.7)	39.0 (36.6–41.3)	56.9 (55.7–58.0)
Both	11.4 (10.8–12.0)	14.7 (13.3–16.2)	10.9 (10.3–11.5)
None	10.2 (9.5–10.8)	10.2 (8.7–11.8)	10.2 (9.5–10.9)

Abbreviation: GED, general education development.

a Percentages are weighted and may not add up to 100% due to rounding.

b Prevalence of vision impairment varied by sociodemographic characteristic (*P* < .05, χ^2^ test).

c Widowed, divorced, separated, or never married.

d Ratio of the family income to the poverty threshold, based on the US Census Bureau federal poverty thresholds given the family’s size and number of children (https://www.census.gov/data/tables/time-series/demo/income-poverty/historical-poverty-thresholds.html).

**Table 2:** Prevalence and Prevalence Ratio of Self-Reported Cardiovascular Disease and Cardiovascular Disease Risk Factors Among US Adults Aged ≥18 Years, by Vision Status, 2018

Risk factor	Total prevalence, % (95% CI)^a^	Prevalence by vision status, % (95% CI)^a^		PR (95% CI)^b^	aPR (95% CI)^c^
		Vision impairment	No vision impairment		
**Cardiovascular disease^d^**	14.1 (13.5–14.7)	26.6 (24.7–28.6)	12.2 (11.7–12.8)	2.18 (2.00–2.37)	1.65 (1.51–1.80)
**Risk behaviors**					
Current smoking^e^	13.8 (13.2–14.4)	19.5 (17.9–21.1)	13.0 (12.3–13.6)	1.50 (1.37–1.64)	1.40 (1.27–1.53)
Physical inactivity^f^	25.7 (24.7–26.8)	34.6 (32.4–36.9)	24.4 (23.3–25.5)	1.42 (1.33–1.51)	1.14 (1.06–1.22)
Excessive alcohol intake^g^	5.3 (4.9–5.6)	6.0 (5.2–7.1)	5.2 (4.8–5.5)	1.17 (0.99–1.39)	1.29 (1.08–1.53)
**Health conditions**					
Obesity^h^	33.0 (32.2–33.9)	42.4 (40.1–44.7)	31.7 (30.7–32.6)	1.34 (1.26–1.42)	1.28 (1.21–1.36)
Hypertension^i^	31.7 (30.9–32.5)	47.5 (45.2–49.7)	29.3 (28.5–30.2)	1.62 (1.53–1.71)	1.29 (1.22–1.36)
High cholesterol^i^	27.9 (27.1–28.7)	38.5 (36.5–40.6)	26.3 (25.5–27.1)	1.46 (1.38–1.55)	1.21 (1.14–1.29)
Diabetes^i^	10.1 (9.6–10.6)	18.5 (16.8–20.3)	8.9 (8.4–9.3)	2.09 (1.88–2.32)	1.54 (1.38–1.72)

Abbreviations: CVD, cardiovascular disease; PR, prevalence ratio; aPR, adjusted prevalence ratio.

a Percentages are weighted percentages and may not add up to 100% due to rounding.

b Separate logistic regression models were performed to generate prevalence ratios for CVD and each CVD risk factor, comparing the prevalence among those with vision impairment to the prevalence of those without vision impairment.

c Adjusted for age (continuous variable), sex, race and ethnicity, education, marital status, employment status, income-to-poverty ratio, and health insurance status.

d Self-reported CVD ascertained by asking whether respondent has ever been told by a doctor or other health professional that they had any of the following conditions: coronary heart disease, angina/angina pectoris, heart attack/myocardial infarction, stroke, or any kind of heart condition or heart disease.

e Current smoker was defined as those who had smoked more than 100 cigarettes in their lifetime and now smoke every day or some days.

f Physical inactivity was defined as performing <10 min per week of light, moderate, or vigorous leisure-time physical activities.

g Excessive alcohol intake was defined as consuming ≥12 drinks in lifetime and >14 drinks/week in past year (for men) or >7 drinks/week in past year (for women).

h Obesity was defined as a body mass index >30. Body mass index was calculated, using self-reported data, as weight (in kilograms) divided by height (in meters) squared.

i Health conditions were defined as an affirmative response to the question of whether the respondent had ever been told by a doctor or other health professional that they had 1) hypertension or high blood pressure, 2) high cholesterol, or 3) diabetes or sugar diabetes.

**Table 3:** Prevalence of Self-Reported Cardiovascular Disease Risk Factors Among US Adults ≥18 Years, by Vision Status and Age Group, 2018

Risk factor	Total prevalence % (95% CI)^a^	Prevalence by vision status % (95% CI)^a^		PR (95% CI)^b^	aPR (95% CI)^c^
		Vision impairment	No vision impairment		
**Cardiovascular disease^d^**					
18–44 y	5.4 (4.9–6.1)	13.4 (10.5–16.9)	4.7 (4.2–5.3)	2.85 (2.19–3.72)	2.53 (1.95–3.28)
45–64 y	14.1 (13.1–15.1)	25.2 (22.3–28.3)	12.0 (11.1–13.0)	2.10 (1.83–2.41)	1.78 (1.55–2.05)
≥65 y	33.8 (32.4–35.3)	43.4 (40.0–46.7)	31.7 (30.2–33.3)	1.37 (1.25–1.49)	1.38 (1.25–1.53)
**Risk behaviors**					
Current smoking^e^					
18–44 y	14.3 (13.4–15.3)	20.7 (17.5–24.4)	13.7 (12.8–14.7)	1.51 (1.27–1.80)	1.36 (1.14–1.62)
45–64 y	16.3 (15.2–17.4)	24.4 (21.6–27.5)	14.7 (13.7–15.8)	1.66 (1.45–1.89)	1.40 (1.22–1.59)
≥65 y	8.6 (7.8–9.4)	10.9 (9.0–13.2)	8.1 (7.3–8.9)	1.35 (1.09–1.68)	1.22 (0.97–1.53)
Physical inactivity^f^					
18–44 y	20.3 (19.0–21.7)	25.5 (21.5–30.0)	19.8 (18.5–21.2)	1.29 (1.09–1.53)	1.10 (0.92–1.31)
45–64 y	26.5 (25.0–28.0)	34.8 (31.6–38.2)	24.9 (23.4–26.5)	1.40 (1.26–1.55)	1.15 (1.04–1.28)
≥65 y	36.9 (35.3–38.6)	44.2 (40.7–47.8)	35.3 (33.6–37.1)	1.25 (1.15–1.37)	1.16 (1.05–1.28)
Excessive alcohol intake^g^					
18–44 y	5.4 (4.9–6.0)	7.1 (5.4–9.4)	5.3 (4.7–5.8)	1.35 (1.01–1.81)	1.45 (1.08–1.94)
45–64 y	5.7 (5.1–6.3)	6.5 (5.3–8.1)	5.5 (4.9–6.2)	1.19 (0.93–1.51)	1.29 (1.02–1.65)
≥65 y	4.3 (3.7–4.9)	4.1 (3.0–5.7)	4.3 (3.7–5.0)	0.97 (0.68–1.38)	1.02 (0.72–1.46)
**Health conditions**					
Obesity^h^					
18–44 y	30.6 (29.3–31.9)	41.4 (36.8–46.2)	29.6 (28.2–30.9)	1.40 (1.24–1.58)	1.33 (1.19–1.50)
45–64 y	37.3 (35.9–38.7)	47.2 (43.8–50.6)	35.4 (33.9–37.0)	1.33 (1.23–1.45)	1.26 (1.16–1.37)
≥65 y	31.7 (30.3–33.0)	36.4 (33.2–39.8)	30.6 (29.1–32.2)	1.19 (1.07–1.32)	1.18 (1.05–1.32)
Hypertension^i^					
18–44 y	12.1 (11.3–13.0)	22.2 (18.6–26.2)	11.2 (10.4–12.0)	1.99 (1.66–2.39)	1.82 (1.52–2.19)
45–64 y	39.5 (38.2–40.9)	50.6 (47.2–54.1)	37.5 (36.0–38.9)	1.35 (1.25–1.46)	1.24 (1.15–1.34)
≥65 y	63.4 (62.0–64.7)	70.7 (67.6–73.6)	61.7 (60.2–63.2)	1.15 (1.09–1.20)	1.16 (1.09–1.23)
High cholesterol^i^					
18–44 y	9.7 (9.0–10.5)	15.2 (12.2–18.9)	9.2 (8.5–10.0)	1.66 (1.32–2.09)	1.60 (1.29–2.00)
45–64 y	36.9 (35.6–38.2)	42.9 (39.5–46.5)	35.8 (34.4–37.2)	1.20 (1.10–1.31)	1.16 (1.07–1.27)
≥65 y	54.6 (53.2–56.0)	57.9 (54.4–61.3)	53.9 (52.3–55.4)	1.07 (1.01–1.15)	1.10 (1.02–1.19)
Diabetes^i^					
18–44 y	3.2 (2.8–3.7)	8.7 (6.2–11.9)	2.7 (2.3–3.1)	3.19 (2.23–4.54)	2.71 (1.92–3.82)
45–64 y	12.5 (11.6–13.6)	21.7 (18.8–25.0)	10.8 (9.9–11.8)	2.01 (1.71–2.35)	1.66 (1.41–1.94)
≥65 y	21.7 (20.6–23.0)	24.7 (21.9–27.7)	21.1 (19.8–22.5)	1.17 (1.02–1.34)	1.11 (0.96–1.28)

Abbreviations: aPR, adjusted prevalence ratio; CVD, cardiovascular disease; PR, prevalence ratio.

a Because of rounding, weighted percentages may not add up to 100%. Sample sizes (unweighted) were n = 8,771 for adults aged 18–44 years, n = 7,670 for adults aged 45–64 years, and n = 6,449 for adults aged ≥65 years.

b Separate logistic regression models with STATA’s *adjrr* command were performed to generate prevalence ratios for CVD and each CVD risk factor, comparing the prevalence among those with vision impairment to the prevalence of those without vision impairment. Each model contained an interaction term between vision impairment and age group to test effect modification by age.

c Adjusted for sex, race and ethnicity, education, marital status, employment status, income-to-poverty ratio, and health insurance status.

d Self-reported cardiovascular disease ascertained by asking whether respondent has ever been told by a doctor or other health professional that they had any of the following conditions: coronary heart disease, angina/angina pectoris, heart attack/myocardial infarction, stroke, or any kind of heart condition or heart disease.

e Current smoker was defined as those who had smoked more than 100 cigarettes in their lifetime and now smoke every day or some days.

f Physical inactivity was defined as performing <10 min per week of light, moderate, or vigorous leisure-time physical activities.

g Excessive alcohol intake was defined as consuming ≥12 drinks in lifetime and >14 drinks/week in past year (for men) or >7 drinks/week in past year (for women).

h Obesity was defined as a body mass index >30. Body mass index was calculated, using self–reported data, as weight (in kilograms) divided by height (in meters) squared.

i Health conditions were defined as an affirmative response to the question of whether the respondent had ever been told by a doctor or other health professional that they had 1) hypertension or high blood pressure, 2) high cholesterol, or 3) diabetes or sugar diabetes.

**Table 4:** Percentage Distribution of Adults by Number of Cardiovascular Disease Risk Factors and Vision Status, Study of Cardiovascular Disease Risk Factors in US Adults With Vision Impairment, 2018

No. of risk factors	Total prevalence % (95% CI)^a^	Prevalence by vision status % (95% CI)^a^	
		Vision impairment	No vision impairment
0	28.0 (27.1–28.9)	16.0 (14.3–17.9)	29.8 (28.9–30.7)
1	29.2 (28.5–30.0)	22.8 (21.0–24.8)	30.2 (29.4–31.0)
2	21.4 (20.7–22.1)	23.9 (22.0–25.9)	21.0 (20.3–21.7)
3	12.7 (12.2–13.2)	19.3 (17.6–21.1)	11.7 (11.2–12.2)
4–7	8.7 (8.3–9.2)	18.0 (16.4–19.7)	7.4 (6.9–7.8)

a Because of rounding, weighted percentages may not add up to 100%. Sample size (unweighted) was N = 22,890.

## Discussion

Our analysis of this nationally representative sample of US adults showed that respondents with VI had a higher prevalence of CVD than those without VI. Approximately 1 in 4 adults with VI reported a CVD diagnosis; approximately 1 in 10 of respondents without VI reported a CVD diagnosis. This finding was consistent with that of a previous study (7). We also found that after adjusting for sociodemographic factors, adults with VI had a higher prevalence of all 7 CVD risk factors that were examined. Furthermore, the relationship between VI and the outcomes of CVD and several CVD risk factors was stronger in the younger age groups. Additionally, more than half of adults with VI reported having 2 or more CVD risk factors (vs 40% among those without VI). Our study adds to existing literature on the relationship between VI and CVD risk factors and strengthens the evidence by examining this relationship among a nationally representative sample of adults aged 18 years or older. Additionally, our study measured general VI, whereas most studies examined CVD risk factors and selected age-related eye diseases (13,14), thereby excluding those who may have VI from other forms of eye conditions.

Prior studies examining VI and CVD risk factors have investigated associations between specific types of eye disease and individual CVD risk factors such as AMD and smoking or glaucoma and hypertension (13–17). For example, one population‐based, cross‐sectional study examining the association of CVD risk factors and AMD found a strong association between current daily smoking and AMD — a leading cause of vision loss for people aged 50 years or older (18). This finding is consistent with our finding that, compared with adults without VI, those with VI had a 40% higher likelihood of being a current smoker. The same study also found sex differences in the association between late AMD (the most severe form of this eye disease) and CVD risk factors. Although the association between AMD and smoking was significant among both men and women, only women had a significant association between late AMD and current smoking, and the same was true for the relationship between late AMD and other CVD risk factors such as obesity, hypertension, and physical inactivity (18). A proposed explanation for this finding is that women generally have a longer life expectancy than men and are therefore more likely to have a longer duration of smoking and greater likelihood of progressing to late-stage AMD, a condition which significantly affects the central vision needed for activities of daily living (19).

Our findings also demonstrate that adults with VI had a 29% higher likelihood of reporting excessive alcohol intake when compared with people without VI. Because our study used cross-sectional data, we were unable to establish temporality or causality for this relationship. However, a longitudinal study examining the relationship of smoking, alcohol consumption, and physical activity to changes in vision over a 20-year period found that people with heavy alcohol consumption had 2.66 times greater odds of incident VI compared with those with occasional alcohol consumption (20). Other studies have reported contradictory results on the associations of alcohol consumption and eye disease, and additional research could elucidate the effects of alcohol on the risk of VI (21). The relationship between CVD and alcohol consumption is complex; however, heavier consumption has generally been associated with negative CVD outcomes. A study investigating health problems associated with alcohol consumption found that CVD was among the most common diseases linked to alcohol consumption, particularly heavy drinking (22). It found that although the overall effect of alcohol consumption on CVD was detrimental, the dose–response relationship differed for different conditions. For example, hypertension risk had a linear relationship with alcohol consumption, indicating an almost entirely detrimental effect. However, for heart disease the association with alcohol consumption showed a J-shaped curve, indicating some protective effects with regular light drinking. This finding is consistent with other studies that have found health benefits to moderate alcohol consumption and an increased risk of illness and death with excessive alcohol consumption (23–25).

One expected finding of our study was that the largest effect size was for the CVD risk factor of diabetes; when compared with adults without VI, those with VI had a 54% higher likelihood of having diabetes. However, the cross-sectional data we used allow only for an assessment of correlation, not causation. Diabetes may have preceded VI, as 1 in 3 people with diabetes will develop diabetic retinopathy, a potentially vision-threatening condition (26). Other studies have shown significant associations between diabetes, poor glycemic control, and other vision-damaging conditions such as glaucoma and cataracts (27). Our study demonstrates a relationship between vision health, diabetes, and CVD health, which is consistent with a recent study that used data from the National Health and Nutrition Examination Survey to examine the association between ideal cardiovascular health and ocular diseases among US adults (5). The study found that 84% of participants with diabetic retinopathy were observed to have inadequate cardiovascular health and that a 1-unit increase in the LS7 ideal cardiovascular health score reduced the odds of diabetic retinopathy by 31% (5). Because of the connection between cardiovascular health and diabetic retinopathy risk, it is important for health care professionals to coordinate CVD management and diabetes care to prevent worsening of chronic disease and increased risk of VI.

Our results showed that 3 in 5 people with VI had multiple CVD risk factors. The 2 most prevalent risk factors among those with VI were hypertension and obesity, with more than 2 in 5 reporting hypertension and nearly 1 in 2 reporting obesity. One US study using nationally representative data found that the odds of having obesity were 1.5 times higher among people with blindness or low vision than the general population (28). Physical activity has been well established as a preventive measure for various chronic diseases including CVD (29). However, engaging in traditional physical activities may be difficult for people with VI. In fact, our study found that adults with VI were more likely to be physically inactive compared with those without VI, although it is unknown whether their activity level preceded VI. Providing physical activity opportunities and health promotion activities for adults with VI is vital to improve health outcomes among this population because evidence has shown that people with VI often have higher rates of poorer health, including overweight and obesity (28). Although this need has been recognized, most health promotion interventions have focused on low-intensity and balance activities for older adults (29). Data on evidence-based health promotion interventions tailored for younger, working-aged adults with VI are limited (29). In addition to tailored lifestyle interventions, clinical intervention could play a key role in preventing disease progression among people with VI. For example, an ophthalmology report reviewing smoking and VI found that advice on smoking cessation from eye care providers increased the odds of quitting smoking by 30% (30).

### Limitations

Our findings are subject to several limitations. First, NHIS consists of self-reported data and can be subject to recall and reporting bias. Second, due to the cross-sectional design of NHIS, causality cannot be established. Third, because NHIS-measured dietary data were not collected in 2018, as they are only collected every 5 years through a sponsored module, we could not use the exact LS7 factors that influence cardiovascular health. We instead used other self-reported CVD risk factor data, such as alcohol consumption, obesity, and diabetes, as proxies for LS7’s diet, body weight, and blood glucose cardiovascular health metrics, respectively. Lastly, although NHIS is nationally representative, it is only administered to noninstitutionalized adults, thus excluding those living in long-term care facilities or institutional settings where the prevalence of VI and chronic health conditions tends to be higher than that in the general population.

### Conclusions

Our results show that adults with VI had a higher prevalence of CVD and CVD risk factors compared with those without VI. The relationship between VI and several CVD risk factors was stronger in the younger age group, demonstrating the potential benefits of early effective clinical and lifestyle interventions, adapted to accommodate VI-related disability to aid in reducing CVD risk in adults with VI. Furthermore, because this association could be bidirectional, integrating vision health into routine clinical care and chronic disease prevention into routine vision services could be beneficial in the prevention and management of CVD and VI.
